# Supplementary material for: Multi‐dataset identification of innovative feature genes and molecular mechanisms in keratoconus
Source: J Cell Mol Med. 2024 Sep 19;28(18):e70079. doi: 10.1111/jcmm.70079 (PMC11412914; doi:10.1111/jcmm.70079)
Supplement: Supplementary file 2 — Material S2. [file JCMM-28-e70079-s002.zip › 7.GSEA analysis/6.SNORA74B/KEGG_GLUTATHIONE_METABOLISM.html]

Details for gene set KEGG\_GLUTATHIONE\_METABOLISM[GSEA]

|  || Dataset | SNORA74B.group.cls#h\_versus\_l |
| Phenotype | group.cls#h\_versus\_l |
| Upregulated in class | l |
| GeneSet | KEGG\_GLUTATHIONE\_METABOLISM |
| Enrichment Score (ES) | -0.4068023 |
| Normalized Enrichment Score (NES) | -1.3881998 |
| Nominal p-value | 0.14343435 |
| FDR q-value | 0.33949706 |
| FWER p-Value | 0.91 |
Table: GSEA Results Summary

  

Fig 1: Enrichment plot: KEGG\_GLUTATHIONE\_METABOLISM      
 Profile of the Running ES Score & Positions of GeneSet Members on the Rank Ordered List

  

| SYMBOL | TITLE | RANK IN GENE LIST | RANK METRIC SCORE | RUNNING ES | CORE ENRICHMENT || 1 | GGT6 | na | 5104 | 0.222 | -0.1049 | No |
| 2 | GGT5 | na | 5963 | 0.193 | -0.0929 | No |
| 3 | GCLM | na | 7816 | 0.141 | -0.1195 | No |
| 4 | GSTA5 | na | 10429 | 0.085 | -0.1787 | No |
| 5 | GSTM5 | na | 10847 | 0.077 | -0.1760 | No |
| 6 | G6PD | na | 11309 | 0.070 | -0.1760 | No |
| 7 | GSTZ1 | na | 12554 | 0.052 | -0.2019 | No |
| 8 | GSTT1 | na | 13556 | 0.041 | -0.2229 | No |
| 9 | MGST2 | na | 14399 | 0.031 | -0.2413 | No |
| 10 | GSTT2 | na | 15264 | 0.022 | -0.2620 | No |
| 11 | GSTA2 | na | 15407 | 0.020 | -0.2622 | No |
| 12 | GGT7 | na | 15680 | 0.018 | -0.2667 | No |
| 13 | GPX1 | na | 16065 | 0.014 | -0.2751 | No |
| 14 | MGST3 | na | 16452 | 0.010 | -0.2843 | No |
| 15 | GSTO2 | na | 17679 | -0.002 | -0.3192 | No |
| 16 | GSTP1 | na | 17706 | -0.003 | -0.3195 | No |
| 17 | GSS | na | 17902 | -0.005 | -0.3242 | No |
| 18 | GSTK1 | na | 17937 | -0.005 | -0.3241 | No |
| 19 | GPX7 | na | 18486 | -0.012 | -0.3377 | No |
| 20 | GPX6 | na | 18772 | -0.015 | -0.3432 | No |
| 21 | OPLAH | na | 19364 | -0.021 | -0.3561 | No |
| 22 | GGT1 | na | 19814 | -0.026 | -0.3640 | No |
| 23 | GPX2 | na | 19981 | -0.028 | -0.3635 | No |
| 24 | GSTO1 | na | 20484 | -0.034 | -0.3715 | No |
| 25 | GCLC | na | 21052 | -0.041 | -0.3801 | No |
| 26 | SMS | na | 21979 | -0.052 | -0.3970 | Yes |
| 27 | TXNDC12 | na | 22089 | -0.053 | -0.3900 | Yes |
| 28 | GPX3 | na | 22223 | -0.054 | -0.3835 | Yes |
| 29 | GPX4 | na | 22406 | -0.057 | -0.3779 | Yes |
| 30 | IDH2 | na | 22633 | -0.060 | -0.3730 | Yes |
| 31 | PGD | na | 23575 | -0.074 | -0.3860 | Yes |
| 32 | GGCT | na | 23648 | -0.075 | -0.3738 | Yes |
| 33 | LAP3 | na | 23824 | -0.078 | -0.3640 | Yes |
| 34 | RRM1 | na | 24063 | -0.082 | -0.3553 | Yes |
| 35 | GSTA3 | na | 24632 | -0.092 | -0.3541 | Yes |
| 36 | GPX5 | na | 24686 | -0.093 | -0.3380 | Yes |
| 37 | GSTM3 | na | 25400 | -0.105 | -0.3385 | Yes |
| 38 | IDH1 | na | 25567 | -0.109 | -0.3226 | Yes |
| 39 | SRM | na | 25596 | -0.109 | -0.3027 | Yes |
| 40 | ANPEP | na | 25605 | -0.109 | -0.2821 | Yes |
| 41 | GSTA4 | na | 25792 | -0.113 | -0.2660 | Yes |
| 42 | GSR | na | 27244 | -0.145 | -0.2802 | Yes |
| 43 | RRM2B | na | 28962 | -0.194 | -0.2928 | Yes |
| 44 | ODC1 | na | 29591 | -0.212 | -0.2705 | Yes |
| 45 | GSTM1 | na | 30369 | -0.240 | -0.2472 | Yes |
| 46 | GSTM2 | na | 32654 | -0.364 | -0.2437 | Yes |
| 47 | GSTM4 | na | 32823 | -0.378 | -0.1766 | Yes |
| 48 | RRM2 | na | 33643 | -0.470 | -0.1108 | Yes |
| 49 | MGST1 | na | 34567 | -0.746 | 0.0046 | Yes |
Table: GSEA details [plain text format]

  

Fig 2: KEGG\_GLUTATHIONE\_METABOLISM      
 Blue-Pink O' Gram in the Space of the Analyzed GeneSet

  

Fig 3: KEGG\_GLUTATHIONE\_METABOLISM: Random ES distribution      
 Gene set null distribution of ES for **KEGG\_GLUTATHIONE\_METABOLISM**
